# Effect of Irreversible Compression on the Pulmonary Nodule Detection Rate in Chest Radiographs Using AI Software

**DOI:** 10.3390/diagnostics16111637

**Published:** 2026-05-27

**Authors:** Masasuke Kohzai, Shintaro Yamamoto, Mika Matsushita, Yutaka Ueno, Noboru Tanigawa

**Affiliations:** Department of Radiology, Kansai Medical University, 2-5-1 Shinmachi, Hirakata 573-1010, Osaka, Japan; yamamosh@hirakata.kmu.ac.jp (S.Y.); matsushita.mik@kmu.ac.jp (M.M.); ueno.yut@kmu.ac.jp (Y.U.); tanigawa.nbr@kmu.ac.jp (N.T.)

**Keywords:** irreversible compression, pulmonary nodule, chest radiographs, artificial intelligence

## Abstract

**Objectives**: Whereas irreversible compression of Digital Imaging and Communications in Medicine (DICOM) files can reduce data size, research on its impact on diagnostic ability when using artificial intelligence (AI) software is limited. The objective was to determine the effect that irreversible compression has on diagnostic ability when using AI software. In addition, the effect of nodal properties on computed tomography (CT) on detection rates was examined. **Methods**: A total of 335 patients with pulmonary nodules were included. Chest radiographs were subjected to irreversible compression at 10:1 and 50:1 ratios. The associations between the detection rate of the AI software and factors such as location on CT, morphology, and diameter, were determined. **Results**: The number of positive cases identified with the AI imaging software was as follows: 188 cases (56.1%) with no compression, 184 cases (54.9%) with 10:1 compression, and 175 cases (52.2%) with 50:1 compression. There was a significant difference between the uncompressed images and the 50:1 compressed images, as well as between the 10:1 compressed images and the 50:1 compressed images (all *p* < 0.05). With all compression ratios, there were significant differences in the associations between the AI software’s nodule detection rate and the target nodule’s maximum diameter, minimum diameter, morphology, and overlap with multiple organs on CT (all *p* < 0.0001). **Conclusions**: The detection rate by the AI software of lung tumors on chest radiographs showed no significant difference when images were subjected to 10:1 irreversible compression; however, there was a significant decrease when subjected to 50:1 irreversible compression.

## 1. Introduction

Chest radiography is one of the most widely used imaging modalities in clinical practice because of its cost-effectiveness, accessibility, and rapid acquisition time. It plays an essential role in the detection and diagnosis of thoracic diseases, including pulmonary nodules, tuberculosis, and interstitial lung diseases. Despite its widespread use, the detection of pulmonary nodules on chest radiographs remains challenging, even for experienced radiologists, due to the superimposition of anatomical structures such as ribs, pulmonary vessels, and mediastinal components. These overlapping structures can obscure subtle lesions and lead to missed diagnoses [1].

In recent years, artificial intelligence (AI)-based computer-aided detection (CAD) systems have been increasingly introduced into clinical workflows to support radiologists in detecting pulmonary nodules [2,3,4,5,6,7,8,9,10,11,12]. Several studies have demonstrated that AI-assisted detection can improve sensitivity and reduce oversight errors, particularly in high-volume screening environments [2,10,13,14,15]. These AI systems are commonly integrated into picture archiving and communication systems (PACS), enabling automated image analysis and decision support [8,12].

However, the integration of AI into radiological workflows also raises practical challenges, particularly regarding data storage and transmission. High-resolution DICOM images require substantial storage capacity and bandwidth, which can limit efficiency in telemedicine, cloud-based analysis, and large-scale screening programs [8,12,16]. Image compression is therefore widely used to mitigate these challenges. While lossless (reversible) compression preserves all original image information, it achieves only modest reductions in file size. In contrast, lossy (irreversible) compression can significantly reduce file size by discarding image information that is presumed to be less important.

Previous studies have evaluated the effects of lossy compression on human visual interpretation of chest radiographs [17,18,19,20,21]. These studies generally suggest that moderate compression ratios, such as 10:1, do not significantly impair diagnostic performance, whereas higher compression ratios, such as 50:1 or greater, may degrade image quality and reduce lesion detectability [17,18,19,20]. However, to date, there has been limited investigation into how lossy compression affects the performance of AI-based CAD systems, which rely on image features that may differ from those used in human perception. Recent reports have suggested that image degradation may affect AI-based image analysis and post-processing algorithm [6,7,22].

Accordingly, the present study aimed to evaluate the impact of irreversible DICOM compression on the performance of an AI-based CAD system for pulmonary nodule detection in chest radiographs. We hypothesized that moderate compression would not significantly affect AI performance, whereas high compression ratios would result in a measurable decline in detection accuracy. By addressing this question, we sought to provide evidence-based guidance for optimizing image compression strategies in AI-assisted radiology workflows.

## 2. Materials and Methods

**Figure 1:**
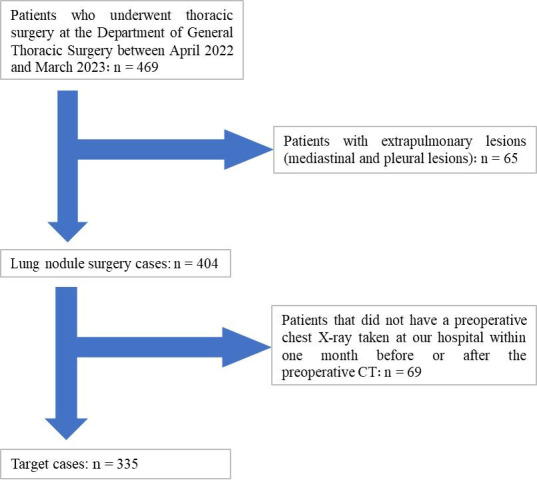
Study flowchart.

This retrospective study was approved by the institutional review board of Kansai Medical University on 14 April 2023, Approval number 2023109, and the requirement for informed consent was waived. The study population consisted of patients who underwent thoracic surgery at our institution between April 2022 and March 2023. A total of 469 consecutive patients were initially identified. Among these, 69 patients were excluded because they did not have a chest radiograph performed within one month before or after preoperative computed tomography (CT), and 65 patients were excluded because their lesions were extrapulmonary, such as mediastinal or pleural lesions. After these exclusions, 335 patients were included in the final analysis (Figure 1).

It should be noted that this study population represents a surgical cohort, which introduces an inherent selection bias. All included patients had nodules that were sufficiently suspicious to warrant surgical resection, resulting in a high prevalence of malignancy. Therefore, the findings of this study may not be directly generalizable to screening populations, where lesion prevalence is lower and nodules are often smaller and less conspicuous.

Chest radiographs were acquired using a digital radiography system (Philips DigitalDiagnost, Philips, Amsterdam, The Netherlands) in the standing posteroanterior position. Imaging parameters included a tube voltage of 120 kVp and a tube current of 1–2 mAs. The images had a matrix size of approximately 2500–3000 × 3000 pixels, providing high spatial resolution. Using the PACS system (Vue PACS, Philips), three versions of each image were generated: uncompressed images, images compressed at a ratio of 10:1, and images compressed at a ratio of 50:1. Compression was performed using a wavelet-based algorithm, which reduces image data by selectively discarding high-frequency spatial information. This is particularly relevant for AI-based analysis, as deep learning models rely on multi-scale feature extraction, including fine edge and texture information.

All images were analyzed using a commercially available AI software designed for pulmonary nodule detection (EIRL X-ray Lung Nodule, version 1.13.1, LPIXEL Inc., Tokyo, Japan). The AI software was operated at the default decision threshold provided by the vendor. The software outputs detection results in the form of bounding boxes with associated confidence scores; however, for the purposes of this study, detection was evaluated as a binary outcome (detected or not detected).

To ensure objectivity, radiologists who performed the evaluation were blinded to the compression ratios of the images. They were also blinded to the confidence scores generated by the AI system during the matching process with CT images. The correspondence between AI-detected nodules and surgically resected nodules identified on CT was assessed independently by two radiologists, and any discrepancies were resolved by consensus.

A detection was considered a true positive when the location of the AI-detected nodule corresponded to the surgically resected nodule identified on CT. Cases in which the AI failed to detect the target nodule were classified as false negatives. Cases in which the AI detected a lesion that did not correspond to the target nodule were classified as false positives. In patients with multiple nodules, only the largest resected nodule was considered the target lesion for analysis. Smaller nodules were not included in the primary endpoint analysis, which may have led to an underestimation of the AI system’s sensitivity for small lesions.

CT images were reviewed using lung window settings with slice thicknesses ranging from 1 to 7.5 mm. The characteristics of the nodules were assessed, including their location, morphology (ground-glass opacity, part-solid, or solid), and size (maximum and minimum diameters). In addition, the presence or absence of overlap between the nodule and anatomical structures such as the diaphragm, heart, or mediastinum was recorded. A time interval of up to one month between CT and chest radiography was permitted, which may have introduced minor discrepancies in nodule size or morphology.

Statistical analysis was performed using JMP Pro software, version 17.0.0 (JMP Statistical Discovery LLC, Cary, NC, USA). Detection rates and false-positive rates were calculated for each compression ratio, along with 95% confidence intervals. The Bowker test was used to compare paired categorical outcomes across compression levels. Univariate analyses were conducted using chi-square tests for categorical variables and the Mann–Whitney U test for continuous variables. Variables that showed significant associations in univariate analyses were included in multivariable logistic regression models to identify independent predictors of nodule detection. Results of the regression analysis were expressed as odds ratios with 95% confidence intervals and adjusted *p*-values. A *p*-value of less than 0.05 was considered statistically significant.

## 3. Results

The study population consisted of 335 patients, including 198 males and 137 females, with a median age of 73 years (Table 1). Histopathological analysis revealed that 300 cases (approximately 89%) were malignant, while 35 cases were benign (Table 2).

**Figure 2:**
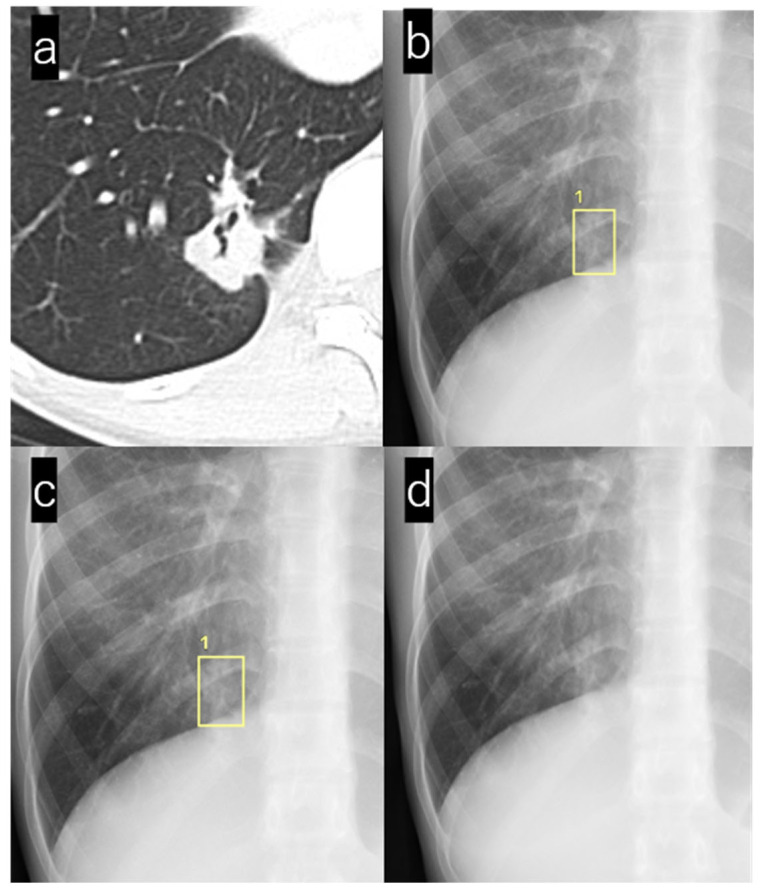
Computed tomography shows a 20 mm nodule in the lower-right lobe of the lung (**a**). The nodule is detected in the full-scale image (**b,1**) and the 10:1 irreversible compression image (**c,1**) when using AI detection of the chest radiograph. It is not detected in the 50:1 irreversible compression image (**d**).

**Figure 3:**
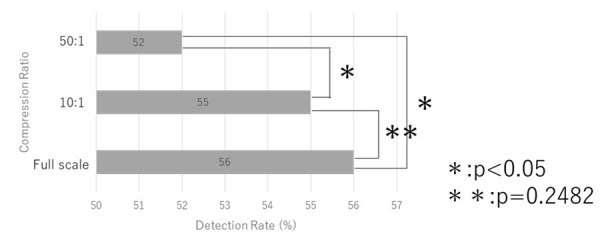
The nodule detection rate of the AI software for each compression ratio. The Bowker test shows significant differences between the uncompressed images and the 50:1 compressed images, as well as between the 10:1 compressed images and the 50:1 compressed images (*p* < 0.05).

The detection rates of pulmonary nodules by the AI software were 56.1% (95% confidence interval [CI]: 50.8–61.3%) for uncompressed images, 54.9% (95% CI: 49.6–60.2%) for images compressed at a ratio of 10:1, and 52.2% (95% CI: 46.9–57.5%) for images compressed at a ratio of 50:1. Statistical analysis using the Bowker test revealed no significant difference between uncompressed images and 10:1 compressed images. However, a statistically significant decrease in detection rate was observed when comparing uncompressed images with 50:1 compressed images, as well as when comparing 10:1 compressed images with 50:1 compressed images (Figure 2 and Figure 3).

The false-positive rates were 13% (95% CI: 9.6–16.9%) for uncompressed images, 13% (95% CI: 9.6–16.9%) for 10:1 compressed images, and 10% (95% CI: 7.1–13.7%) for 50:1 compressed images. The reduction in false-positive rates at the higher compression ratio suggests that compression may suppress both signal and noise, resulting in an overall decrease in detection sensitivity.

Subgroup analysis revealed that nodules with larger diameters, solid morphology, and absence of overlap with anatomical structures were more likely to be detected by the AI system. In contrast, nodules with ground-glass opacity morphology demonstrated markedly low detection rates across all compression levels. Specifically, only 8 of 46 ground-glass opacity nodules (17.4%) were detected in uncompressed images, indicating a substantial limitation of the AI system in identifying low-contrast lesions (Table 3).

Multivariable logistic regression analysis confirmed that larger nodule size, solid morphology, and absence of anatomical overlap were independent predictors of detection. Compression at a ratio of 50:1 was independently associated with reduced detection performance, with an odds ratio of 0.78 (95% CI: 0.62–0.98), indicating a statistically significant negative effect.

## 4. Discussion

There have been several reports on the impact of DICOM lossy compression on doctors’ diagnostic performance [17,18,19,20,21], but there have been no reports on its impact on AI diagnostic performance. Therefore, this issue was studied.

In the present study, the nodule detection rate of an uncompressed image was 56.1%.

Many studies have reported nodule delineation in chest radiographs using AI [2,3,4,5,6,7,8,9,10,11,12,13,14,15]. Sim et al. reported that combining a radiologist’s diagnosis with AI software increased the detection sensitivity of lung nodules by 5.2% [13]. The detection sensitivity of lung nodules of the AI software itself performed by their multiple teams was 67.3% (56.1% to 82.7%).

When Majkowska et al. conducted a study examining two datasets, they reported that radiologists and AIs have comparable diagnostic abilities regarding chest radiographs when it comes to detecting pneumothorax, increased density, nodules, and fractures [14]. The nodule sensitivity of AI software was 82.4% and 44.1% in each respective dataset.

According to Nam et al., a higher nodule detection rate was found in the AI group than in the non-AI group when examining chest radiographs from health checkups [2]. The detection rate of malignant lung nodules was significantly higher in the AI group; however, caution is needed in interpreting these results due to the very low incidence of the disease. Recent review articles have highlighted the expanding role of deep learning in chest radiograph interpretation and thoracic oncology, while also emphasizing concerns regarding robustness, hidden stratification, and generalizability in AI systems [6,7,14,15].

To the best of our knowledge, there have been no reports of AI being used on compressed images; however, several studies have evaluated compressed images with the naked eye [17,18,19,20,21].

In chest radiographs, low compression ratios do not affect the visual evaluation; however, when compression ratios are high, it is known that there is a decrease in the detection rate of findings and a lowering of diagnostic ability due to image deterioration [17,18,19,20]. Ishigaki et al. investigated the detection rate of nodular shadows and linear opacity of chest radiographs subjected to irreversible compression and reported that 10:1 compression is acceptable [17]. Richard et al. compared uncompressed images with images that had been compressed at ratios of 10:1, 20:1, and 50:1 [20]. Image deterioration and the presence or absence of the appearance of artifacts were confirmed, and it was reported that the 10:1 and 20:1 compressed images were visually lossless. However, loss of fidelity was notable in the 50:1 compressed images [18]. MacMahon et al. found that 25:1 irreversible compression related to lung nodules, interstitial opacity, bone lesions, and pneumothorax did not result in a significant reduction in diagnostic ability; however, a decrease in accuracy was notable in the 50:1 compressed images [18]. Savcenko et al. observed no differences in diagnostic accuracy and detection ability for calcified lung nodules and fibrosis between uncompressed images and images that were irreversibly compressed at a 40:1 ratio [19]. They also noted that, although there is a decrease in detection ability at 80:1 compression, there is no difference in diagnostic accuracy [20]. A visual examination of the 10:1 compressed image showed no effect on the detection rate or diagnostic ability due to image deterioration when compared to an uncompressed image. However, compression ratios higher than 50:1 are considered to affect the detection rate and diagnostic ability.

In the present study, the detection rate of lung nodules in chest radiographs using AI software was significantly lower in 50:1 compressed images than in uncompressed images, but there was no significant difference in 10:1 compressed images. The ability of AI software to detect nodules in compressed images was comparable to the visual evaluation of compressed images. In the present study, when cases that tested negative in the analysis by the AI software were examined using CT, nodules that were small in size, had no solid components, and were overlapping with multiple organs tended not to be detected by the AI software. The same was reported in previous studies that examined compressed images through visual evaluation [6,7,8,9]. The decrease in nodule detection rates in compressed images may be attributed to the difficulty in delineating nodule margins, caused by the decreased image quality due to the reduced pixel count.

This study has several limitations. The first limitation was that the target cases were extracted from surgical cases. Surgical cases include not only cases with abnormalities identified on chest radiographs but also many cases in which abnormalities were observed only on CT. The second limitation was the size of the nodules that the AI software used in the present study was able to handle. The AI software used in the present study was designed to detect nodule sizes between 5 mm and 3 cm, and there is the possibility that anything larger or smaller may not be detected. The third limitation was the fact that this was a retrospective study with a limited number of cases. There is a need for further investigation through large-scale prospective studies in the future.

## 5. Conclusions

The 10:1 compressed images are comparable to the full-size images for the pulmonary nodule detection by AI software analysis, but 50:1 compressed images are not suitable. Compression ratios need to be taken into consideration when using AI software for irreversibly compressed images.

## Figures and Tables

**Table 1:** Participants’ characteristics.

Patients	335
Males/Females	198/137
Age, y (median, range)	73, 21–88
Benign/Malignant	35/300
Maximum diameter, mm (median, range)	18, 3–120
Minimum diameter, mm (median, range)	13, 2–100
Overlap with multiple organs (Yes/No)	253/82
Morphology (GGO/part-solid/solid)	46/42/247
Location, right (right upper, right middle, right lower)	208
Right upper lobe	105
Right middle lobe	18
Right lower lobe	85
Location, left (left upper, left lower)	127
Left upper lobe	65
Left lower lobe	62

GGO = Ground-glass opacity.

**Table 2:** Tumor histopathology.

Benign Tumor	35	Malignant Tumor	300
Inflammatory granuloma	5	Adenocarcinoma	156
Amyloid tumor	4	Squamous cell carcinoma	48
Aspergilloma	3	Lung metastasis	47
Organizing pneumonia	3	Large intestine	29
Hamartoma	2	Pancreas	3
		Kidney	2
		Thyroid glands	2
		Salivary glands	2
		Bladder	2
		Liver	2
		Others	5
Necrotizing nodule	2	MIA	9
Sarcoidosis	2	Small cell cancer	8
Ciliated muconodular papillary tumor/Bronchiolar adenoma	1	Pleomorphic carcinoma	7
Fibroelastosis	1	Adenosquamous cancer	5
Interstitial pneumonia	1	AIS	4
Bronchiectasis	1	IMA	3
Giant cell granuloma	1	Malignant lymphoma	3
Hematoma	1	Carcinoid	2
Sclerosing pneumocytoma	1	AAH	1
Postoperative scars	1	Lymphoepithelial carcinoma	1
Fibrous scars	1	Sarcomatoid carcinoma	1
Post-treatment scars	1	Choriocarcinoma	1
Granuloma	1	Neuroendocrine cancer	1
Lung abscess	1	Large cell neuroendocrine cancer	1
Non-tuberculous mycobacterial infection	1	Large cell cancer	1
Right middle lobe syndrome	1	Lymphoproliferative disorder	1
		Carcinoma	1

MIA = minimally invasive adenocarcinoma (MIA), AIS = adenocarcinoma in situ, IMA = invasive mucinous adenocarcinoma, AAH = atypical adenomatous hyperplasia. “Large intestine” to “Others” are all categories of “lung metastasis“.

**Table 3:** CT findings and detection rates.

Full-Scale Image (n = 335)	Positive (n = 188)	Negative (n = 147)	*p*-Value
Sex (M:F)	112:76	86:61	0.9109
Age, years (median, range)	72, 21–88	74, 40–87	0.195
Benign/Malignant	21/167	14/133	0.7199
Maximum diameter, mm (median, range)	22, 6–103	14, 3–120	<0.001
Minimum diameter, mm (median, range)	15, 4–58	11, 2–100	<0.001
Morphology (GGO/part-solid/solid)	8/27/153	38/15/94	<0.001
Location (right upper, right middle, right lower, left upper, left lower)	65/7/47/42/27	40/11/38/23/35	0.0508
Overlap with multiple organs	24%	59%	<0.001
			
**10:1 (n = 335)**	**Positive (n = 184)**	**Negative (n = 151)**	***p*** **-value**
Sex (M:F)	108:76	90:61	0.9113
Age, y (median, range)	72, 21–88	74, 45–87	0.1208
Benign/Malignant	19/165	16/135	1
Maximum diameter, mm (median, range)	22, 6–103	14, 3–120	<0.001
Minimum diameter, mm (median, range)	15, 4–58	11, 2–100	<0.001
Morphology (GGO/part-solid/solid)	7/26/151	39/16/96	<0.001
Location (right upper, right middle, right lower, left upper, left lower)	64/7/44/40/29	41/11/41/25/33	0.1626
Overlap with multiple organs	14%	66%	<0.001
			
**50:1 (n = 335)**	**Positive (n = 175)**	**Negative (n = 160)**	***p*** **-value**
Sex (M:F)	102:73	96:64	0.824
Age, years (median, range)	72, 36–88	74 68–79	0.1298
Benign/Malignant	21/154	14/146	0.3742
Maximum diameter, mm (median, range)	22, 6–103	14, 3–120	<0.001
Minimum diameter, mm (median, range)	15, 3–58	11, 2–100	<0.001
Morphology (GGO/part-solid/solid)	7/24/144	39/18/103	<0.001
Location (right upper, right middle, right lower, left upper, left lower)	62/8/41/37/27	43/10/43/28/35	0.2506
Overlap with multiple organs	11%	70%	<0.001

GGO = Ground-glass opacity.

## Data Availability

The original contributions presented in this study are included in the article.

## Abbreviations

The following abbreviations are used in this manuscript:

DICOM	Digital Imaging and Communications in Medicine
AI	artificial intelligence
CAD	computer-aided detection
PACS	picture archiving and communication system
FOV	field of view
GGO	ground-glass opacity
MIA	minimally invasive adenocarcinoma
AIS	adenocarcinoma in situ
IMA	invasive mucinous adenocarcinoma
AAH	atypical adenomatous hyperplasia
